# Impact of C-reactive protein and albumin levels on short, medium, and long term mortality in patients with diffuse large B-cell lymphoma

**DOI:** 10.1080/07853890.2022.2046287

**Published:** 2022-03-03

**Authors:** Kim Oren Gradel, Thomas Stauffer Larsen, Henrik Frederiksen, Pernille Just Vinholt, Maria Iachina, Pedro Póvoa, Fernando Godinho Zampieri, Stig Lønberg Nielsen, Ram Benny Dessau, Jens Kjølseth Møller, Thøger Gorm Jensen, Ming Chen, John Eugenio Coia, Jelena Jelicic

**Affiliations:** a Center for Clinical Epidemiology, Odense University Hospital, and Research Unit of Clinical Epidemiology, Department of Clinical Research, University of Southern Denmark, Odense C, Denmark; b OPEN – Odense Patient Data Exploratory Network, Odense University Hospital, Odense C, Denmark; c Department of Haematology, Odense University Hospital, and Research Unit of Haematology, Department of Clinical Research, University of Southern Denmark, Odense C, Denmark; d Department of Clinical Biochemistry and Pharmacology, Odense University Hospital, Odense C, Denmark; e The Polyvalent Intensive Care Unit, Hospital de São Francisco Xavier, CHLO, Estrada do Forte do Alto do Duque, Lisbon, and NOVA Medical School, CEDOC, New University of Lisbon, Lisbon, Portugal; f Research Institute, dHCor-Hospital Do Coração, São Paulo, Brazil; g Department of Infectious Diseases, Odense University Hospital, and Research Unit of Infectious Diseases, Department of Clinical Research, University of Southern Denmark, Odense, Denmark; h Department of Clinical Microbiology, Slagelse Hospital, Slagelse, Denmark; i Department of Clinical Microbiology, Vejle Hospital, University Hospital of Southern Denmark, Vejle, Denmark; j Department of Clinical Microbiology, Odense University Hospital, and Department of Clinical Research, Research Unit of Clinical Microbiology, University of Southern Denmark, Odense C, Denmark; k Department of Clinical Microbiology, Hospital of Southern Jutland, Sønderborg, Denmark; l Department of Clinical Microbiology, Hospital of South West Jutland, Esbjerg, Denmark

**Keywords:** Diffuse large B-cell lymphoma, prognosis, C-reactive protein, plasma albumin, inflammation

## Abstract

**Objectives and study design:** In this population-based study of 602 patients, we amended C-reactive protein (CRP) and plasma albumin (PA) levels around the diagnosis of diffuse large B-cell lymphoma (DLBCL) to the International Prognostic Index (IPI) and assessed 0–90, 91–365, and +365-day survival.

**Results:** The CRP did not contribute to the IPI's prognostic or discriminatory ability, regardless of time period, particularly not in models with PA. In contrast, the PA was an important contributor, especially in the 0–90 day period, but also up to one year after the diagnosis. For day 0–90, the model with the IPI only had an Area Under the Receiver Operating Characteristics (AUROC) of 0.742, whereas the IPI with PA as a continuous variable rendered an AUROC of 0.841. Especially the lower PA quartile (18–32 g/L) contributed to the worse prognosis.

**Conclusions:** The amendment of PA to the IPI may significantly improve the short-term prognostic and discriminative ability.

**Key messages**
- The amendment of the plasma albumin (PA) level to the International Prognostic Index significantly improved the prediction of mortality up to one year after the diagnosis of diffuse large B-cell lymphoma.
- It was especially the lower quartile of the PA level (18–32 g/L) that contributed to the worse prognosis.

## Introduction

Diffuse large B cell lymphoma (DLBCL) is the most common type of lymphoma, accounting for about 30–40% of all non-Hodgkin lymphomas [1]. Approximately two-thirds of the patients can be cured with a standard first line therapy, but the remaining third will eventually develop a relapsed/refractory disease, which is the major cause of morbidity and mortality in patients with DLBCL. In an attempt to stratify patients into various risk groups and in order to identify high-risk patients, several prognostic indices have been developed and validated for DLBCL [2]. The most widely used index [2] is the International Prognostic Index (IPI), which is based on patient and cancer related factors (age, ECOG performance score [3], Ann Arbour stage, extra-nodal involvement, and lactate dehydrogenase concentration) [4]. Newer indices, such as the National Comprehensive Cancer Network IPI (NCCN-IPI) [5], have been suggested as more discriminative, especially after the introduction of Rituximab. However, these prognostic indices do not capture lymphoma related molecular aberrations causing particularly aggressive high-risk disease, such as cell of origin, activated B-cell type DLBCL, MYC and/or BCL2 and/or BCL6 gene re-arranged or double expressor lymphomas [6]. Nor do they capture the potential impact of the tumour microenvironment, including host immune reactions and inflammatory processes.

The role of inflammation in cancer [7], including the prognostication of solid malignancies [8], has received much attention in recent years. One of the more well-established and non-molecular inflammatory indices, which is easy to use in the clinic, is the Glasgow Prognostic Score (GPS) [9]. The GPS is based on C-reactive protein (CRP) and plasma albumin (PA) levels, both of which are inflammatory markers [10,11]. Of note, few prognostic studies of hematological malignancies have incorporated inflammatory markers in spite of several studies showing their impact on the pathogenesis of lymphomas [12,13]. To our knowledge, seven studies have included both CRP and PA in the prognostication of DLBCL [14–20], but only one assessed whether these contributed to the discriminatory ability of globally accepted prognostic DLBCL indices [17].

We thus wished to elucidate whether CRP and/or PA contribute to the prognostic and discriminative ability of the IPI [4] in patients with DLBCL [2]. We evaluated CRP and PA individually and together, and as continuous or categorical variables to deduce their impact on the overall survival (OS) and progression-free survival (PFS), in different periods (0–90, 91–365, and >365 days after the diagnosis).

## Materials and methods

### Setting

The Danish public health system is tax-financed, and consequently free of charge for the individual patient [21]. All patients with lymphoma are treated in specialised haematology departments in public hospitals. Geographically well-defined catchment areas for these hospitals enable population-based studies. The unique Danish identification number, used in all registries, enables the merging of data [21].

### Study cohort

From the Danish National Lymphoma Registry (LYFO) [22] we retrieved all adult patients (16 years or older) with first-time diagnosed DLBCL, admitted to Odense University Hospital from 2000 through 2017. LYFO has high positive predictive values (87–100%) and completeness (92–100%), using the Danish Cancer Registry and the Danish National Patient Registry as gold standards [23] and it comprises numerous prospectively recorded clinical variables, including the IPI [4]. The LYFO data were merged with biochemistry parameters from 2000 through 2017, vital status as per 24 November 2017, and bloodstream infection (BSI) episodes derived from microbiological data [24].

Among the 907 patients diagnosed with DLBCL, we included the 602 who had a non-missing IPI, both CRP and PA measured ±10 days within their day of diagnosis (D0), and information on vital status (one patient excluded because he emigrated before D0) for the final study cohort.

### Analyses of CRP and PA levels

CRP was measured with an immune-turbidimetric principle and PA by use of a bromocresol green dye-binding method, both on modular P® (Roche, Mannheim, Germany).

### Statistical analyses

We used the CRP and PA levels closest to D0 within the ±10-day period in all the analyses.

Initially, we computed baseline patient characteristics (e.g. sex, age, IPI distribution) in contingency tables and compared these by Chi-square or t-test statistics, as appropriate.

We computed quartiles from the CRP/PA levels and related the distribution of these quartiles to the four mortality risk scores of the IPI (low, low/intermediate, high/intermediate, high).

Overall survival (OS) was defined as the time from D0 until death, regardless of cause [25] and progression-free survival (PFS) as the time from D0 until disease progression, relapse, or death (regardless of cause) [26]. For OS, we computed Kaplan-Meier curves for the CRP and PA quartiles and the IPI, covering the first year after D0. After assessing graphically that the proportional hazard assumptions were fulfilled we then computed Cox’s regression analyses with hazard ratios (HRs) and 95% confidence intervals (CIs) to determine OS and PFS. We divided the follow-up period into 0–90, 91–365, and >365 days after D0. The follow-up time ceased when the outcome occurred or the patient was censored (emigration, the upper limit (day 90 or 365) was encountered, or 24 November 2017 [last recorded date of the vital status] occurred), whichever came first. We defined the baseline model as the four scores of the IPI. We amended the CRP and PA levels to the baseline model, separately and together, categorised in quartiles and as continuous variables. We further dichotomised the CRP (cut-off 10 mg/L) and PA levels (cut-off 35 g/L) to compute the GPS [9] and amended this to the baseline model.

To assess the impact of each covariate in each of the three time periods, we computed and scatter plotted the HRs for the combinations of the four IPI scores, PA quartiles, and CRP quartiles, using the model with OS as outcome.

Based on the Cox’s regression analysis results, we derived areas under the receiver operating characteristic curves (AUROCs) [27] and used these to assess the discriminatory ability of the different models and compared them by C-statistics [28].

Many of the patients had BSIs, which *per se* had an impact on the CRP and PA levels. To deduce whether this would have an impact on the overall results we therefore reiterated all analyses with the exclusion of patients who had a BSI episode ±30 days in relation to D0.

To further assess the robustness of our results we reiterated all the analyses with the NCCN-IPI instead of the IPI as the baseline model.

In a final sub-analysis we only included the 517 patients (85.9%) who were treated with immunochemotherapy/Rituximab-based therapy (R-CHOP/R-CHOP-like).

A p-value <0.05 was considered statistically significant. The program Stata®, vs. 16, (StataCorp., College Station,TX, USA) was used for all analyses.

### Ethical considerations

According to Danish legislation, no approval from an ethics committee or consent from participants is required for registry-based studies. Because biochemical and microbiological data were derived from legal medical record data, permission from the Danish Patient Safety Authority (rec. no. 3–3013-2019/1) was obtained. The project was listed in the Region of Southern Denmark’s record (rec. no. 21/15491).

## Results

### Baseline characteristics

Amongst the final study cohort’s 602 patients, 39.5% were females, the age range was 16.5–90.7 years, and the median age was 67.1 years (Table 1). Twenty-one patients (3.5%) had DLBCL, which was only detected in the central nervous system. The four IPI scores were evenly distributed. Nearly one fifth had at least one BSI episode, one-year mortality was 24.6%, and 18.6% had relapsed/refractory DLBCL.

**Table 1.** Characteristics of 907 patients with diffuse large B-cell lymphoma (DLBCL).

Text	Study cohort^a^	Excluded patients^b^
	(*n* = 602)	(*n* = 305)
Females	238 (39.5)^c^	134 (43.9)
Age		
Range, years	16.5–90.7	15.0–96.2
Median (inter-quartile range), years	67.1 (57.2–75.1)	69.8 (61.2–77.7)
International Prognostic Index		
Low	157 (26.1)^d^	79 (38.0)
Low/intermediate	127 (21.1)	44 (21.2)
High/intermediate	171 (28.4)	55 (26.4)
High	147 (24.4)	30 (14.4)
Unknown	0 (−)	97 (−)
Bloodstream infections (from D–30)^e^	117 (19.4)	57 (18.7)
One episode	91 (15.1)	39 (12.8)
More than one episode	26 (4.3)	18 (5.9)
± 30 days within date of diagnosis of DLBCL	36 (6.0)	15 (4.9)
Treatment		
CHOP^f^/CHOP-like, Rituximab	517 (85.9)	249 (81.6)
CHOP/CHOP-like	59 (9.8)	39 (12.8)
HDMTX^g^, +/− Rituximab	21 (3.5)	12 (3.9)
Palliative	5 (0.8)	5 (1.6)
Deceased		
Day 0–90	55 (9.1)	34 (11.2)
Day 91–365	93 (15.4)	42 (13.8)
Beyond day 365	135 (22.4)	89 (29.2)
Unknown^h^	10 (1.7)	5 (1.6)
Relapsed/refractory DLBCL		
Day 0–90	7 (1.2)	0 (0)
Day 91–365	53 (8.8)	20 (6.6)
Beyond day 365	52 (8.6)	37 (12.1)

^a^Non-missing International Prognostic Index, both C-reactive protein and plasma albumin measured ±10 days within their day of diagnosis, and information on vital status.

^b^Patients who did not fulfil one or more of the criteria listed in table note 1.

^c^Number (%), except for age, cf. text.

^d^‘Unknown’ excluded in computation of percentages as the International Prognostic Index was one of the inclusion criteria.

^e^One or more bloodstream infection episodes from 30 days before date of diagnosis of DLBCL and onwards.

^f^Cyclophosphamide, Hydroxydaunorubicin, Oncovin, Prednisone/Prednisolone.

^g^High-dose Methotrexate.

^h^Patients diagnosed with DLBCL <365 days before the last date of the vital status (24 November 2017). Time between date of diagnosis and 24 November 2017 ranged from 210 through 353 days for the study cohort and from 312 through 359 days for 4 excluded patients (moreover, 1 patient was excluded due to emigration before his date of diagnosis).

The 305 excluded patients with missing results (CRP, PA, IPI, or vital status) were 3.2 years older (*p* = 0.001) and had lower IPI scores (*p* = 0.002), but the remaining characteristics were similar to those of the study cohort (Table 1).

### Distributions of CRP and PA quartiles in relation to the IPI

The distributions of the quartiles between the four IPI scores were uneven, both for CRP and PA (Figure S1). In the low IPI score, 116/158 (73.4%) CRP levels were found in the two lower quartiles, but this gradually shifted with increasing IPI score, with the two upper quartiles contributing with 106/147 (72.1%) in the high IPI score. For PA, we saw the reverse trend, 116/158 (73.4%) in the two upper quartiles in the low IPI score, whereas the two lower quartiles contributed with 97/147 (66.0%) in the high IPI score. Overall, the results of both CRP and PA suggested that increasing inflammation was associated with an adverse risk profile.

### Kaplan-Meier curves for OS, up to one year after D0

**Figure 1.**
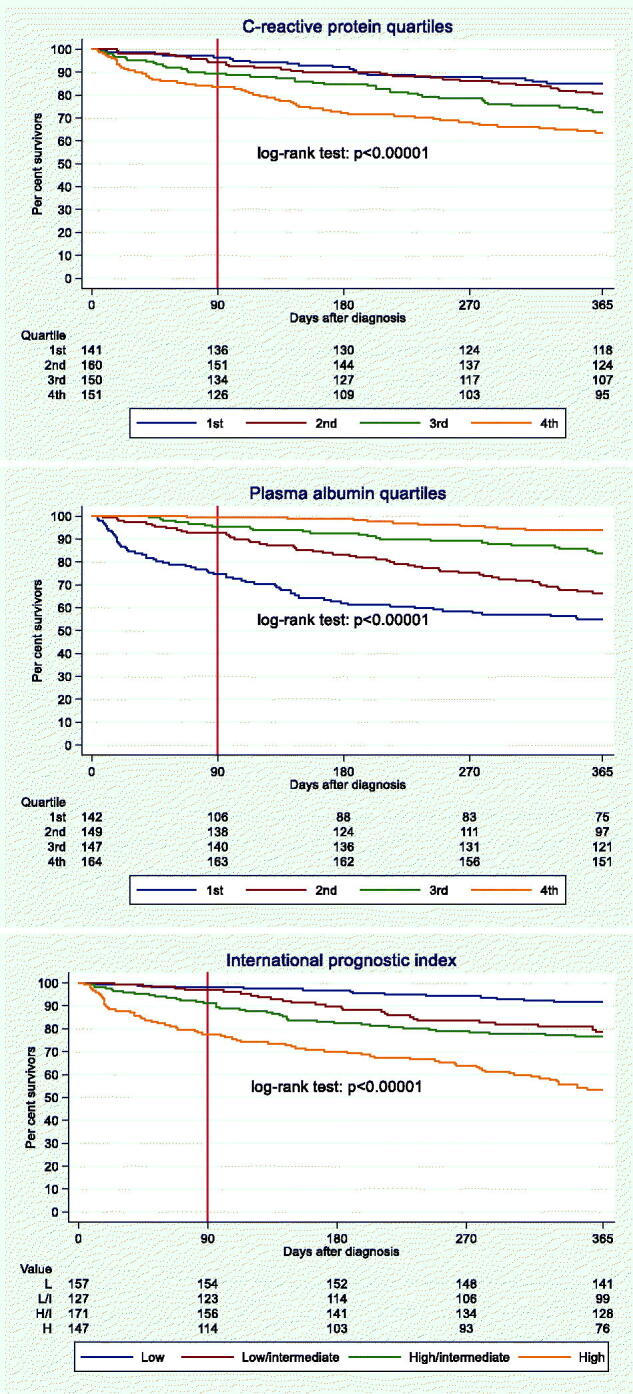
Kaplan-Meier survival curves, 0–365 days, for C-reactive protein quartiles, plasma albumin quartiles, and International Prognostic Index scores.

Increasing CRP quartile (Figure 1, upper panel) and decreasing PA quartile (Figure 1, middle panel) were both associated with a worse prognosis. The CRP quartiles depicted smaller survival differences than the PA quartiles. On day 90, it was mainly the lower PA quartile that deviated from the three other PA quartiles as well as from the CRP quartiles, but on day 365 all four PA quartiles were clearly deviating more than the four CRP quartiles. In total, 14.9% in the lower and 36.4% in the upper CRP quartile (factor 2.4 [36.4/14.9]) died within one year. As for PA, 45.1% of the patients in the lower quartile vs. 6.1% in the upper quartile died (factor 7.4).

The separation of the Kaplan-Meier curves for the four IPI scores (Figure 1, lower panel) resembled those of the curves for PA quartiles (Figure 1, middle panel).

### Cox’s regression analyses

The 602 patients had 971,602 follow-up days, median (IQR) 1079 (347–2,398) days, and a range of 4–6,465 days.

For OS as outcome, the baseline model showed a clear trend of higher HRs with increasing IPI score in the 0–90 day period, which diminished in the 91–365 day period, and vanished beyond 365 days (Table 2). These trends were the same when CRP and PA quartiles were amended to the model, but the HRs declined, especially in the 0–90 day period. The same was seen when CRP and PA as continuous variables or the GPS were amended (data not shown). Regardless of time period, CRP quartiles were not associated with OS. There was a clear trend of lower mortality with increasing PA quartile in the first two periods whereas the only difference beyond day 365 was seen between the lower and the three remaining quartiles.

**Table 2.** Cox’s regression analyses for overall survival.

Text	0–90 days		91–365 days		>365 days	
	Baseline model^a^	Baseline model with CRP^b^ and PA^c^ quartiles	Baseline model	Baseline model with CRP and PA quartiles	Baseline model	Baseline model with CRP and PA quartiles
International Prognostic Index						
Low	Reference	Reference	Reference	Reference	Reference	Reference
Low/intermediate	1.65 (0.37–7.36)^d^	1.21 (0.27–5.45)	3.09 (1.47–6.49)	2.75 (1.30–5.81)	1.61 (1.01–2.58)	1.66 (1.03–2.68)
High/intermediate	4.73 (1.37–16.3)	2.30 (0.65–8.18)	2.67 (1.28–5.57)	1.96 (0.92–4.16)	1.68 (1.07–2.65)	1.50 (0.92–2.43)
High	13.2 (4.06–43.2)	6.05 (1.77–20.6)	5.34 (2.64–10.8)	3.64 (1.74–7.62)	1.74 (1.03–2.95)	1.52 (0.85–2.72)
CRP quartile (mg/L)						
1 (0–4.8)	–	Reference	–	Reference	–	Reference
2 (5–24)	–	0.77 (0.25–2.38)	–	0.90 (0.47–1.73)	–	0.98 (0.60–1.59)
3 (25–79)	–	0.74 (0.26–2.15)	–	0.66 (0.34–1.29)	–	0.89 (0.52–1.54)
4 (80–433)	–	0.97 (0.34–2.73)	–	0.83 (0.43–1.61)	–	0.78 (0.44–1.38)
PA quartile (g/L)						
1 (18–32)	–	Reference	–	Reference	–	Reference
2 (33–37)	–	0.30 (0.15–0.59)	–	1.06 (0.65–1.74)	–	0.54 (0.32–0.92)
3 (38–41)	–	0.20 (0.09–0.47)	–	0.41 (0.22–0.77)	–	0.62 (0.37–1.04)
4 (42–52)	–	0.04(0.01–0.32)	–	0.21 (0.09–0.48)	–	0.47 (0.27–0.84)

^a^The model that includes the International Prognostic Index only.

^b^C-reactive protein.

^c^Plasma albumin.

^d^Hazard ratio (95% confidence interval).

The results with PFS as outcome did not deviate materially from those of OS, except that the HRs for the IPI scores in the first two periods were smaller (Table S1).

### Scatter plots of HRs vs. Combinations of IPI scores, PA quartiles, and CRP quartiles

**Figure 2.**
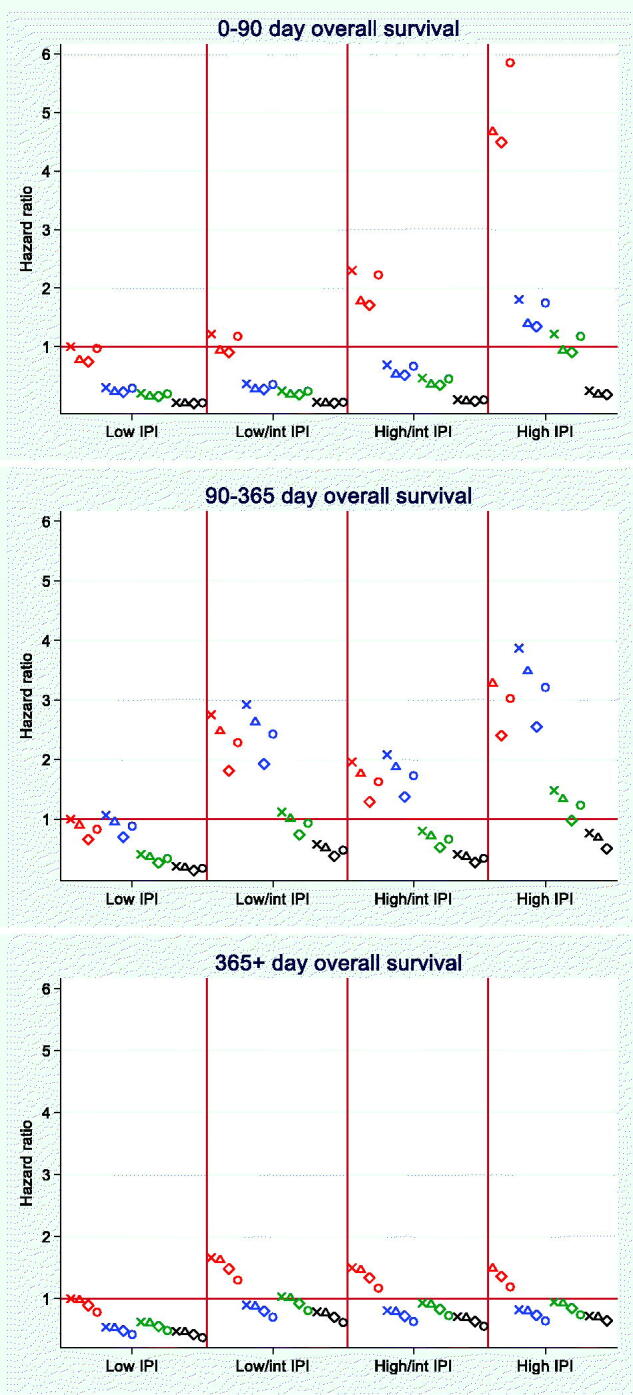
Mortality rate ratios of combinations of International Prognostic Index (IPI) scores (low, low/intermediate, high/intermediate, high), plasma albumin quartiles (red: lower quartile [18–32 g/L]; blue: 2^nd^ quartile [33–37 g/L]; green: 3^rd^ quartile [38–41 g/L]; black: upper quartile [42–52 g/L]), and C-reactive protein quartiles (X: lower quartile [0–4.8 mg/L]; hollow triangle: 2^nd^ quartile [5–24 mg/L]; hollow diamond: 3^rd^ quartile [25–79 mg/L]; hollow circle: upper quartile [80–433 mg/L]).

As each subgroup included a low number of patients, these results only depict general patterns and CIs have consequently been omitted from the HRs in Figure 2.

In the 0–90 day period, it was especially the lower PA quartile that deviated from the other three quartiles, a deviation that increased with higher IPI score (Figure 2, upper part). In the low IPI score, no HRs were above 1, whereas only patients with PA in the lowest quartile had HR >1 in the low/intermediate and the high/intermediate IPI scores. Only in the high IPI score did we encounter HRs >1 for the three lower, but not for the highest PA quartile. HRs for the CRP quartiles were very close to each other within each PA quartile.

In the 91–365 day period, it was especially the two lower PA quartiles that had HR >1 and these HRs increased with higher IPI score (Figure 2, middle part). The CRP quartiles were still close to each other within the PA quartiles, but a trend towards lower HR with higher CRP quartile within each combination of PA quartile and IPI score was seen.

Beyond day 365, we saw small differences between the IPI scores, but higher HRs were especially encountered for the lower PA quartile, regardless of the IPI score (Figure 2, lower part). As for the 91–365 day period, higher CRP quartiles were inversely correlated with the HRs.

### AUROCs for the models

The baseline model had an AUROC of 0.742 for the 0–90 day OS (Table 3). The amendment of CRP slightly increased this AUROC (*p* = 0.03), but the largest increase was seen when the PA was amended to the model as a continuous variable (*p* < 10^−4^), rendering an AUROC of 0.841. The amendment of CRP as a continuous variable did not alter this AUROC (*p* = 0.75). The GPS increased the AUROC to 0.784, which was less than the full models with CRP and PA as separate variables (*p* = 0.001). CRP and PA as continuous variables yielded slightly higher AUROCS than as quartiles, although this was not significant (*p* = 0.14). All AUROCs for the 91–365 day mortality period diminished, but the highest contribution of PA was unaltered. All AUROCs beyond 365 days were close to 0.5 and the models were not significantly different from each other (all p-values 0.08 or higher).

**Table 3.** Areas under the Receiver Operating Characteristics Curve (AUROCs), 0–90, 91–365, and >365 day overall survival.

Model	0–90 days	91–365 days	>365 days
IPI^a^ (Baseline model)	0.742 (0.684–0.799)^b^	0.642 (0.591–0.693)	0.559 (0.507–0.611)
IPI, CRP^c^ quartiles	0.770 (0.717–0.824)	0.657 (0.605–0.708)	0.567 (0.512–0.622)
IPI, PA^d^ quartiles	0.825 (0.781–0.869)	0.711 (0.664–0.758)	0.592 (0.537–0.647)
IPI, CRP and PA quartiles	0.828 (0.784–0.872)	0.717 (0.669–0.765)	0.590 (0.534–0.646)
IPI, CRP as continuous variable	0.768 (0.712–0.825)	0.649 (0.597–0.700)	0.556 (0.503–0.609)
IPI, PA as continuous variable	0.841 (0.798–0.884)	0.707 (0.658–0.756)	0.585 (0.531–0.639)
IPI, CRP and PA as continuous variables	0.841 (0.798–0.884)	0.713 (0.664–0.763)	0.587 (0.533–0.641)
IPI, Glasgow Prognostic score	0.784 (0.733–0.835)	0.672 (0.621–0.722)	0.581 (0.527–0.635)

^a^International Prognostic Index.

^b^AUROC (95% confidence interval) and letter.

^c^C-reactive protein.

^d^Plasma albumin.

AUROCS for the models with PFS as outcome were very similar to those for OS (data not shown).

### Analyses with the exclusion of patients with a BSI episode ±30 days in relation to D0

The exclusion of patients who had one or more BSI episodes from 30 days before through 30 days after D0 did not change the overall results materially, neither for OS (Table S2) nor for PFS (data not shown).

### Analyses with the NCCN-IPI

For 553 patients in our study cohort (91.9%) and 64 of the 305 excluded patients (21.0%) we could compute the NCCN-IPI and reiterate all the analyses. All results were materially the same as in the models encompassing the IPI (data not shown), e.g. an AUROC of 0.856 for the 0–90 day OS in the model with CRP and PA as continuous variables.

### Subgroup analyses in relation to treatment with R-CHOP/R-CHOP-like

Results of all analyses for the 517 patients who received R-CHOP/R-CHOP-like did not deviate materially from the overall results (Table S3 shown for OS).

## Discussion

In patients with DLBCL, we found that inflammation, as assessed by CRP and PA, was prognostically predictive for both OS and PFS, especially in the short-term (0–90 days after the DLBCL diagnosis), but also up to one year after diagnosis. Moreover, inflammatory markers contributed to the discriminatory ability of the well-established IPI, e.g. an increase of the AUROC from 0.74 to 0.84 in the short-term. Finally, we found that low PA values contributed considerably to the prognosis regardless of the IPI score and time period (Figure 2).

The CRP is considered a gold standard marker of inflammation [10], but when PA was amended to the model, CRP had no prognostic impact in our study. This is in line with studies of CRP in other patient groups, e.g. patients with BSI where one-time measurements were weak prognostic markers [29] whereas longitudinal assessments were more predictive [30].

An increasing amount of studies and reviews interprets PA as an inflammatory rather than a nutritional marker [11,31] and hypoalbuminemia is generally considered a strong prognostic predictor, regardless of patient group [32]. Consequently, PA has been incorporated in prognostic indices, such as the APACHE III score for patients in intensive care where the amendment of PA improved its discriminatory ability [33]. As regards indices for hematological malignancies, PA is currently only incorporated routinely in the International Staging System for Myeloma [34] and the International Prognostic Score for advanced Hodgkin lymphoma [35].

The GPS has been assessed as a prognostic index in mostly solid malignancies [9]. As either CRP >10 mg/L or PA <35 g/L leads to one GPS point, each contributes equally to the GPS [36]. Moreover, a modified GPS, in which PA <35 g/L does not contribute to the index if CRP is ≤10 mg/L, also exists [36]. In this study of patients with DLBCL, CRP did not contribute to the short- or long-term prognosis, which was also reflected in the lower AUROC for the model with the GPS as compared to the model with PA. Moreover, the prognostic predictability was strongest for PA as a continuous variable, which further weakens the impact of the GPS, with its dichotomisation of CRP and PA levels [37].

We found 19 prognostic studies that either assessed CRP only [38–40], PA only [41–49], or both [14–20] in patients with DLBCL, most of which amended CRP and/or PA to globally accepted prognostic DLBCL indices [14–20,38–42,44,46,48,49].

However, only four of these studies, all of which assessed mortality without time restrictions, evaluated the discriminatory ability of CRP and/or PA by AUROCs [17,40,44,46]. A study of 1990 Danish patients, also derived from the LYFO registry, showed that the amendment of PA to the IPI increased the AUROC from 0.73 to 0.76 (*p* < 0.0001) [44]. The study focussed more on the specific factors of the IPI, whereas ours mainly dealt with inflammatory markers and consequently we divided the follow-up period into short, medium, and long term mortality. Importantly, for the model with PA as a continuous variable and 0–90 day mortality, we found an AUROC of 0.84 which is considered excellent discrimination [50]. The Danish results were corroborated in an Austrian study where an AUROC of 0.75 in an NCCN-IPI model increased to 0.78 when PA was amended (*p* < 0.0001) [46]. In the same study cohort, the amendment of CRP increased the AUROC of 0.75 to 0.79 (*p* < 0.0001) [40]. The fourth study, from Japan, was the only previous study that evaluated PA and CRP together [17]. The study showed that PA, but not CRP, was associated with higher OS and PFS in a multivariate model, with an AUROC of 0.64 without PA and 0.74 with PA in the model. Our results are in line with all of those four studies, but the division into 0–90, 91–365, and +365 day survival, with decreasing AUROCs, corroborated that inflammation was especially important in the short-term prognosis.

To our knowledge, this is the first study of patients with DLBCL that has assessed CRP and PA separately and together in discriminatory analyses, with both short, medium, and long term mortality as outcomes. We found that both the CRP and the PA levels were relatively stable around D0 (data not shown), which enabled the measurement closest to D0 within a 10-day period. Results were robust and materially the same regardless of whether OS or PFS were used as outcomes, with the exclusion of patients with a BSI from 30 days before through 30 days after D0, for the IPI vs. the NCCN-IPI, and for patients treated with R-CHOP/R-CHOP-like therapy. Data were highly valid [23,51], population-based [21], and comprised a number of patients that enabled robust results. Only 2 of the 19 prognostic studies that evaluated CRP and/or PA for patients with DLBCL included more patients than ours [44,47].

Our study also had limitations that deserve further attention. Firstly, the LYFO registry records the IPI whereas the updated NCCN-IPI may show higher discriminatory ability [5]. Although the IPI was implemented before the introduction of Rituximab in routine management it is still the most commonly used prognostic index for DLBCL [2]. Moreover, because the IPI was assessed prospectively and recorded directly in the LYFO database, whereas we could only compute the NCCN-IPI retrospectively, we think the IPI results are more valid. However, the replacement of the IPI with the NCCN-IPI for a slightly different study cohort did not change the results materially (data not shown). Secondly, the exclusion of patients without PA and CRP retrieved close to time of diagnosis may have introduced selection bias, although we believe this is of minor importance (Table 1). Thirdly, our retrospectively derived data had little information on clinical aspects, e.g. steroid treatment before the DLBCL diagnosis, tumour control, or causes of death. Many of our patients were elderly and may have died due to causes unrelated to their DLBCL status. However, albumin is known to be a strong prognostic predictor regardless of disease or cause of death [52]. Fourthly, we did not have valid data on other infections than BSI. We have previously shown that only 32.3% of BSIs were recorded in the Danish National Patient Registry [53], and we suspect that other, less severe, infections may even be more underreported in administrative registries. It is highly probable that other infections than BSI have triggered high CRP and low PA values, as we have shown for numerous CRP peaks and PA troughs that were unrelated to BSI episodes in patients with acute myeloid leukaemia [24]. Consequently, our hypothesis-generating study cannot elucidate whether the prognostic impact of especially PA was mainly driven by the malignancy *per se* or the frequently accompanying infections.

In conclusion, we found that the amendment of PA around the time of diagnosis to the globally accepted IPI improved its discriminatory ability concerning OS and PFS within the first year after diagnosis. The best model was seen with PA as a continuous variable, but we also found that it was mainly the lower (18–32 g/L) or the two lower quartiles of PA (18–37 g/L) that contributed to the prognosis. Hence, a cut-off level around 36 g/L could be applied in an international prospective validation study with the aim of incorporating it into the IPI. The IPI has been valid in the Rituximab era, but novel therapies, e.g. CAR T-cells, may decrease its value as a prognostic index. In contrast, a one-time level of CRP around the time of diagnosis contributed very little to the prognostic predictability of the IPI.

## Data Availability

According to Danish legislation, access to research data is only possible under the auspices of the registry researcher (KOG) and data will remain stored on a central Danish research server.
